# A two-stage method for automated detection of ring-like endosomes in fluorescent microscopy images

**DOI:** 10.1371/journal.pone.0218931

**Published:** 2019-06-27

**Authors:** Dongyun Lin, Zhiping Lin, Jiuwen Cao, Ramraj Velmurugan, E. Sally Ward, Raimund J. Ober

**Affiliations:** 1 School of Electrical and Electronic Engineering, Nanyang Technological University, Singapore, Singapore; 2 Key Lab for IOT and Information Fusion Technology of Zhejiang, Hangzhou Dianzi University, Hangzhou, China; 3 Department of Biomedical Engineering, Texas A&M University, College Station, TX, United States of America; 4 Department of Molecular and Cellular Medicine, Texas A&M University Health Science Center, College Station, TX, United States of America; 5 Department of Microbial Pathogenesis and Immunology, Texas A&M University Health Science Center, Bryan, TX, United States of America; 6 Cancer Sciences Unit, Centre for Cancer Immunology, Faculty of Medicine, University of Southampton, Southampton, United Kingdom; Duke-NUS Medical School, SINGAPORE

## Abstract

Endosomes are subcellular organelles which serve as important transport compartments in eukaryotic cells. Fluorescence microscopy is a widely applied technology to study endosomes at the subcellular level. In general, a microscopy image can contain a large number of organelles and endosomes in particular. Detecting and annotating endosomes in fluorescence microscopy images is a critical part in the study of subcellular trafficking processes. Such annotation is usually performed by human inspection, which is time-consuming and prone to inaccuracy if carried out by inexperienced analysts. This paper proposes a two-stage method for automated detection of ring-like endosomes. The method consists of a localization stage cascaded by an identification stage. Given a test microscopy image, the localization stage generates a voting-map by locally comparing the query endosome patches and the test image based on a bag-of-words model. Using the voting-map, a number of candidate patches of endosomes are determined. Subsequently, in the identification stage, a support vector machine (SVM) is trained using the endosome patches and the background pattern patches. Each of the candidate patches is classified by the SVM to rule out those patches of endosome-like background patterns. The performance of the proposed method is evaluated with real microscopy images of human myeloid endothelial cells. It is shown that the proposed method significantly outperforms several state-of-the-art competing methods using multiple performance metrics.

## 1 Introduction

**Fig 1:**
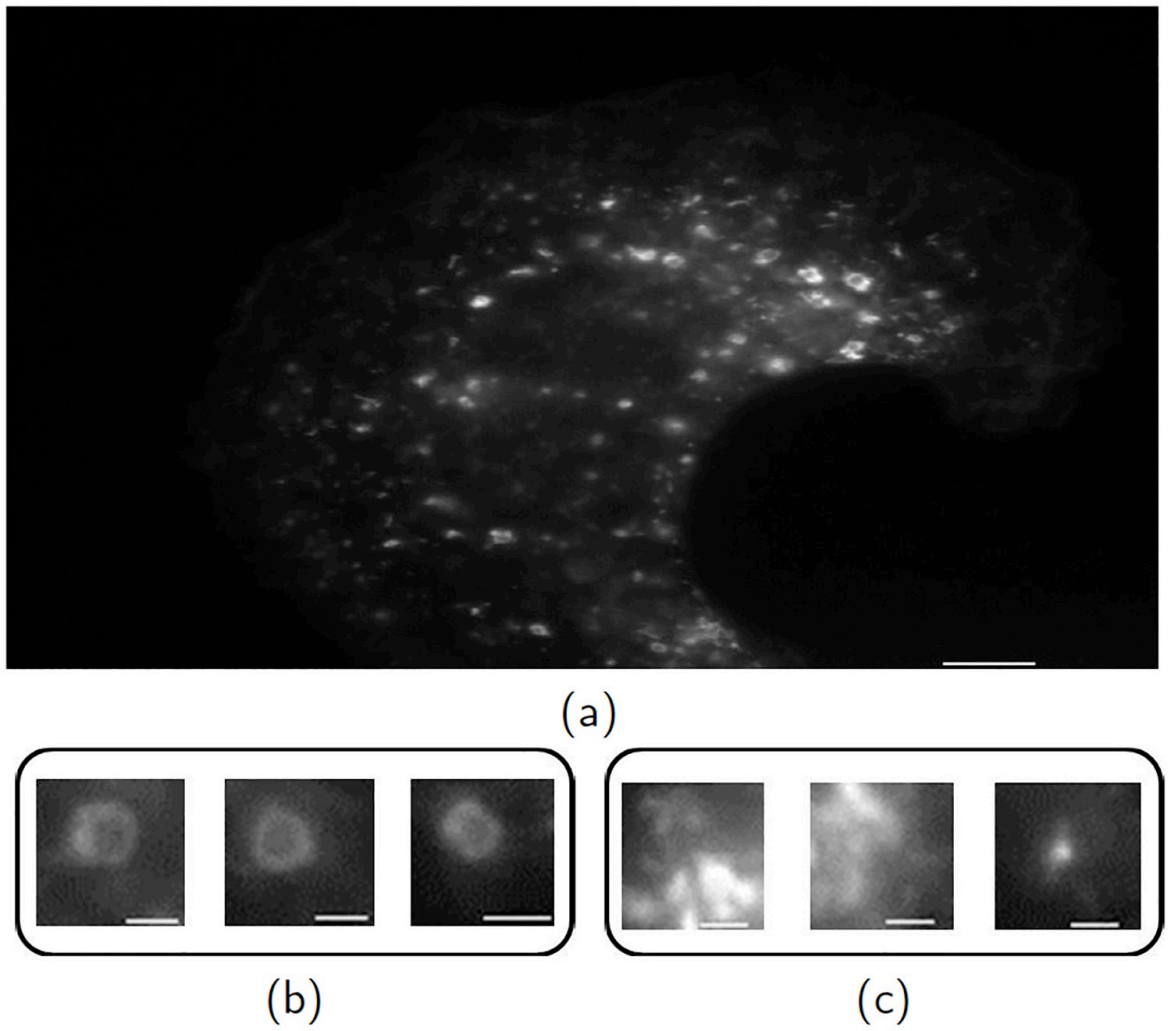
A microscopy images of a cell with multiple subcellular structures. (a) The whole microscopy image. (b) Three patches of ring-like endosomes. (c) Three patches of background patterns. The scale bar for (a) is 5 *μ*m and that for (b) and (c) is 1 *μ*m.

Fluorescent microscopy can produce snapshots of subcellular structures inside cells (e.g., see Fig 1a). Endosomes (shown in Fig 1b) are organelles which can be found in all eukaryotic cells and function as important transport compartments that shuttle proteins, nutrients and other materials inside cells [1]. The detection of ring-like endosomes from background patterns (e.g., see Fig 1c) is of significant biological interest relating to the analysis of interactions among proteins and organelles. For example, ring-like endosomes are relevant to the assessment of the effectiveness of a particular class of drugs called therapeutic monoclonal antibodies [2]. The annotation of endosomes is usually performed manually, which is time-consuming and inaccurate if carried out by inexperienced analysts. Hence, it is valuable to automate the process of endosome detection.

In the last decade, machine learning approaches have achieved great success in addressing tasks related to microscopy images (see e.g., [3–6]). For instance, the structures and functions of different proteins were studied based on their locations in certain organelles using image classification methods [7–12]. Motivated by these publications, this paper aims to propose a machine learning method which can automatically detect ring-like endosomes in microscopy images.

Several methods for detecting subcellular structures in fluorescent microscopy images have been proposed in the literature. Ref. [13] carried out a quantitative evaluation of multiple methods for spot detection. In general, detection methods can be divided into two groups: unsupervised and supervised methods. Unsupervised methods require some prior knowledge of the appearance model of the target and usually contain parameters to be tuned either manually or semi-automatically [13]. For instance, ref. [14] applied the fast radial symmetry transformation (FRST) [15] to detect cell nuclei based on the information that cell nuclei are approximately circular and symmetrical. On the other hand, supervised methods first “learn” the discriminative appearance information of the target from annotated patches, i.e., small regions of interest (ROIs), each of which contains either an isolated target (positive patch) or an irrelevant background pattern (negative patch). Then it searches in the test images for patches of targets from a classification perspective. An example of a supervised method is the Viola-Jones detector, i.e., the Adaboost classifier trained on Haar-like features [16, 17]. In general, most unsupervised methods are computationally efficient and perform well when the targets have simple and distinct features, e.g., distinguishing circular targets from highly non-circular background patterns. However, unsupervised methods normally fail to detect complicated targets especially when the adopted prior information is not sufficiently discriminative to distinguish the targets from the background patterns. In such scenarios, supervised methods generally outperform unsupervised methods as they better exploit the discriminative information between the targets and the background patterns. Therefore, we focus on supervised methods in this paper.

Supervised methods require a training phase before testing. In the training phase, a classifier is trained to discriminate between positive and negative patches. In the testing phase, given a microscopy image, sliding windows, i.e., overlapping small patches which scan over a relatively large image, are adopted to search all the locations in the microscopy image. Each window is fed to the pretrained classifier to determine whether it contains the target or not. In this paper, the aforementioned sliding window based supervised methods are called one-stage methods to distinguish them from our proposed two-stage supervised method. A disadvantage of one-stage supervised methods is the adoption of the sliding window search as it is computationally complicated in general and might miss the detection if the target is partially covered or “mismatched” with the sliding window, e.g., a small target staying inside a relatively large sliding window. Another important limitation of a one-stage supervised method is that the performance of its classifier highly depends on the selection of training patches that can be represented by discriminative feature vectors. Moreover, most one-stage supervised methods only exploit global information of each training patch, i.e., representing a training patch (as a whole) by a feature vector, which might deteriorate the detection performance.

In this paper, we propose a two-stage supervised method for the automated detection of ring-like endosomes in fluorescent microscopy images. The proposed method consists of a localization stage cascaded by an identification stage. Given a test microscopy image, the localization stage generates a voting-map by locally comparing the query endosome patches and the test image based on a bag-of-words model. Using the voting-map, a number of candidate patches of endosomes are determined. Subsequently, in the identification stage, a support vector machine (SVM) is trained from the endosome patches and the background pattern patches. Each of the candidate patches is classified by the SVM to rule out those patches of endosome-like background patterns. Compared with one-stage supervised methods, the proposed method has several advantages. In the localization stage, the proposed method directly localizes potential candidate patches of endosomes using the voting-map based approach, thereby avoiding the computationally complicated sliding window search. Each candidate patch locates a potential endosome at its center and the size of the patch can be automatically determined, which alleviates the “mismatched” effect caused by the sliding window search. Moreover, unlike one-stage methods which only exploit global information of training patches, the proposed method applies the local information of training patches in the localization stage and the global information in the identification stage. Both stages perform complementarily to improve the detection performance. This paper represents a significant development upon our preliminary works reported in [18].

The organization of the remaining parts of this paper is as follows. The dense SIFT based bag-of-words model is first introduced in Section 2.1 as both stages of the proposed method are based on this model. Then, the details of the localization and identification stages are presented in Sections 2.2 and 2.3, respectively. For experiments, Section 3.1 provides the specific information of the data sets. The performance metrics are introduced in Section 3.2. The experimental protocols and the parameter settings for the compared methods are specified in Section 3.3. In Section 3.4, the qualitative and quantitative experimental results are presented and analyzed, respectively. Section 4 concludes the entire paper.

## 2 Materials and methods

The proposed method consists of two stages: localization and identification. The localization stage is designed to provide location information of candidate patches. The identification stage is proposed to identify endosomes by further ruling out those candidate patches containing endosome-like background patterns from a classification perspective. As both stages exploit the dense SIFT based bag-of-words model for image representation, a brief review of it is given first.

### 2.1 Dense SIFT based bag-of-words model

**Fig 2:**
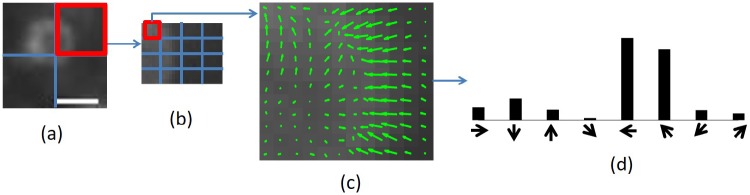
A diagram illustrating the procedure to extract dense SIFT features from an image. (a) Given an image, it is divided into 2 × 2 = 4 grids. (b) Each grid is then divided into 4 × 4 = 16 subgrids. Due to the border effects, the subgrids appeared at the border of each grid might have fewer pixels than the other subgrids. (c) For each subgrid, the magnitude and orientation of the gradient for each pixel are calculated. The green arrows denote the gradients where the length and direction of each arrow are the magnitude and the orientation of the gradient, respectively. (d) A histogram of gradients with respect to 8 orientation bins (in the range of 0 to 360 degrees) is built to summarize the gradients within each subgrid. A gradient is assigned to its nearest orientation bin by comparing the gradient’s orientation with the 8 orientation bins. The weight of the assignment is the magnitude of the gradient. If a gradient has its orientation in the middle of 2 neighboring orientation bins, a bilinear interpolation is applied. By this procedure, each grid shown in (a) can be represented by concatenating 16 8-bin histograms, i.e., a 16 × 8 = 128 dimensional SIFT feature vector. The scale bar of Fig 2(a) is 1 *μ*m.

**Fig 3:**
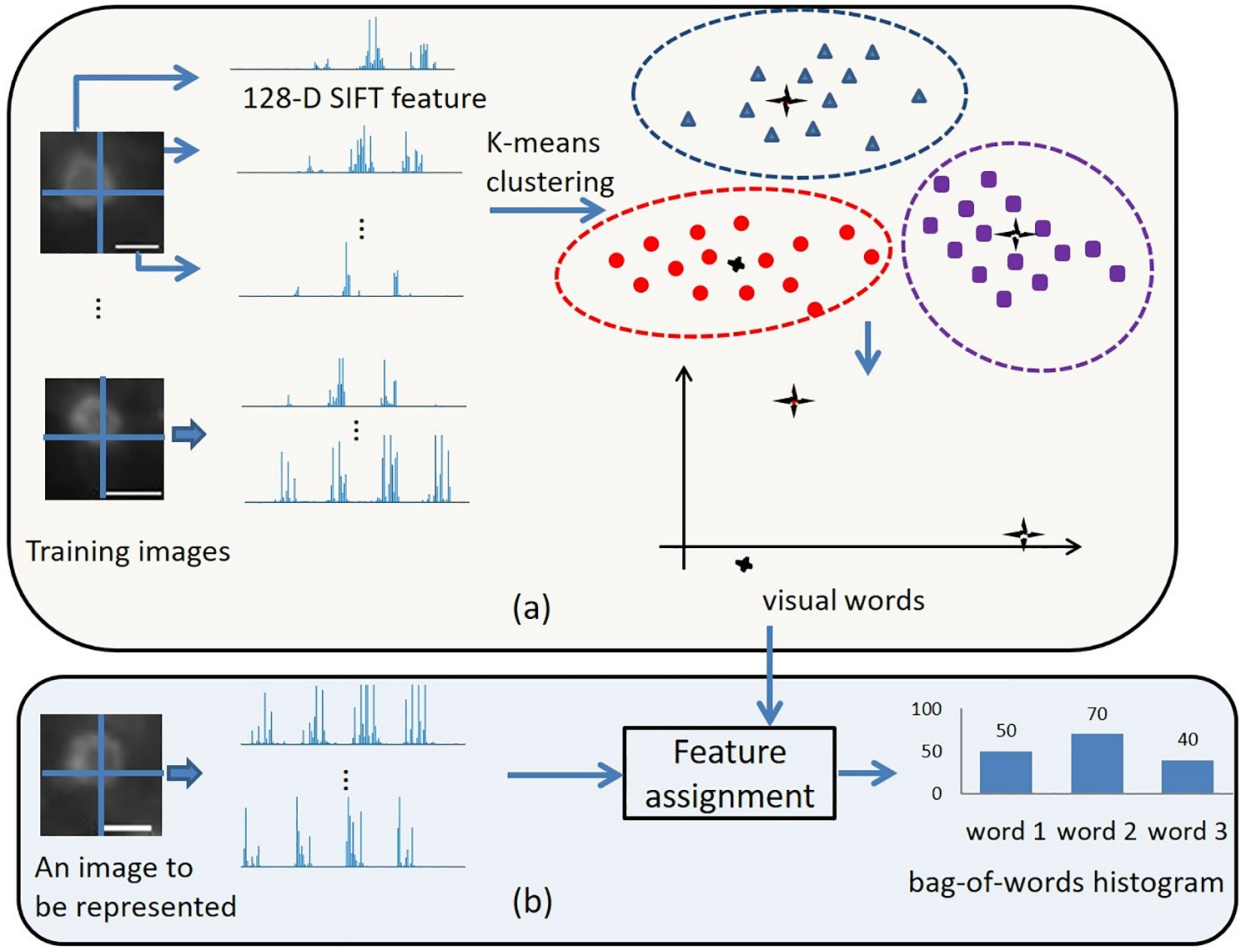
A diagram illustrating how to generate the bag-of-words visual words from several training images and how to represent an image by a bag-of-words histogram associated with these visual words. (a) The training images are decomposed into many SIFT features. By K-means clustering, these SIFT features are clustered to a predefined number of classes. The centroids of these clusters are referred as the visual words. In this example, three visual words are generated. The set containing all the visual words is referred as a dictionary. (b) Given an image to be represented by a bag-of-words model, it is first decomposed into several SIFT features. Each SIFT feature is assigned to its nearest visual word using Euclidean distance, i.e., vector quantization. Implementing such feature assignment for each SIFT feature, the image can be represented as a histogram of visual word frequencies, i.e., the counts of the occurrences of the SIFT features assigned to each visual word. Scale bar = 1*μ*m.

The bag-of-words model is popular in image classification [19, 20] and retrieval [21, 22]. This model determines a set of representative local features (called the visual words) from a number of training images using unsupervised clustering, e.g., K-means clustering. An image can be represented as a bag-of-words histogram associated with the visual words. Here, we extract scale invariant feature transform (SIFT) features [23] from an image. SIFT features are used to describe the micro-structures of microscopy images as they remain robust across noise and illumination variation [23]. Particularly in this work, dense SIFT features are extracted from microscopy images. Unlike the original SIFT approach where a small number of keypoints are first determined from an image and SIFT features are extracted from the grids centering at these keypoints [23], dense SIFT features are extracted from dense and overlapping grids which cover the whole image [19]. Fig 2 illustrates the procedure of extracting dense SIFT features from an image. Based on this procedure, Fig 3 illustrates how to generate the bag-of-words visual words from several training images and how to represent an image by a bag-of-words histogram associated with these visual words. Note that the term “image” in both figures refers to its general meaning and it can mean a “small patch” or a “large microscopy image” depending on the context. Subsequently, note that both Figs 2 and 3 decompose the images into non-overlapping grids for easy illustration. In practice, overlapping grids for dense SIFT feature extraction are implemented.

### 2.2 Localization stage

**Fig 4:**
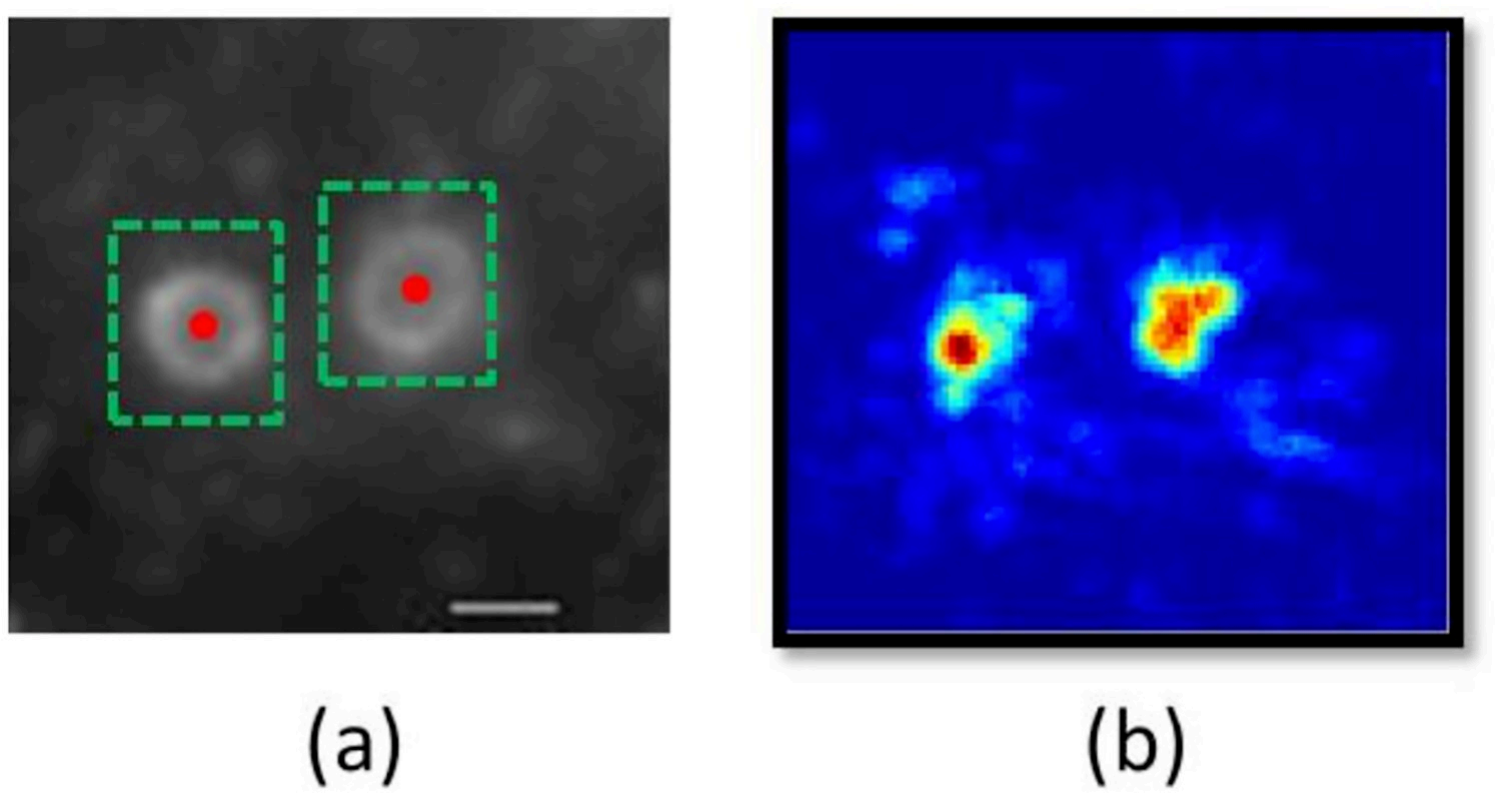
(a) The candidate patch of an endosome in a microscopy image can be determined by the centroid of the endosome (denoted by the red dot) and a rectangle box that contains the endosome (denoted by dashed green rectangles). (b) The voting-map for the microscopy image shown in (a). Scale bar = 1 *μ*m.

The localization stage is proposed to determine candidate patches of endosomes from a microscopy image. Since endosomes are approximately circular with ring-like textures, a candidate patch of an endosome in a microscopy image can be determined by the centroid of the endosome (a pixel in the microscopy image) and a rectangle box that contains the endosome as shown in Fig 4a. To determine the centroids of the candidate endosomes, a voting-map, i.e., an image of the same size as the microscopy image, is generated. Each pixel in the voting-map quantifies the confidence of the centroid of an endosome. Fig 4b shows an example of the voting-map associated with the microscopy image in Fig 4a.

Motivated by the techniques proposed in [21] on natural object localization using one query patch of the object, we propose an improved category localization method for our application using multiple query patches of endosomes. Here, a query patch of an endosome is a patch of a standard endosome selected by hand. In this stage, each of the query patches and the test microscopy image are locally compared to determine the potential locations of endosome centroids. These locations are “voted” by a quantity called discriminative capability [24] to be defined later. After performing such a measurement between every query patch and the test microscopy image, a voting-map is generated. The remaining parts of this subsection show the technical details of the localization stage consisting of a training phase and a testing phase.

#### 2.2.1 Training phase

In the training phase, dense SIFT features are first extracted from the query endosome patches and the background pattern patches. Then, a set of visual words is generated using the SIFT features. Based on the visual words, each patch is represented by a bag-of-words histogram. The location and assignment (to a visual word) for each SIFT feature are recorded. Finally, the discriminative capability of each visual word is determined. These quantities will be adopted in the testing phase for voting-map generation.

Specifically, we manually collect *M* (e.g., *M* = 50) patches of endosomes as a query set denoted by ***Q***_*set*_ = {***Q***_1_, ***Q***_2_, …, ***Q***_*M*_} and another set of *N* (e.g., *N* = 200) patches of background patterns denoted by ***B***_*set*_ = {***B***_1_, ***B***_2_, …, ***B***_*N*_}. The size of each of these patches is of 45 × 45 pixels. The local SIFT features are extracted from each of the patches in ***Q***_*set*_ and ***B***_*set*_. Then a bag-of-words dictionary with *K* (e.g., *K* = 1000) visual words is generated from these SIFT features. With the dictionary, the bag-of-words histograms are calculated for the patches. For each query patch in ***Q***_*set*_, the location of each of its SIFT features, i.e., the center pixel position of the grid where the SIFT is calculated, is recorded.

Subsequently, the discriminative capability [24] is calculated to quantify the significance of each visual word in discriminating the patches of endosomes from those of the background patterns. The discriminative capability ***d***(*k*), *k* = 1, 2, …, *K*, for the *k*_*th*_ visual word is defined as the ratio of the within-class similarity and the between-class similarity as:

$$\begin{array}{c} d\left(k\right)=\frac{\alpha (k)}{\delta (k)}. \end{array}$$ (1)

The within-class similarity ***α***(*k*) associated with the endosome class (***Q***_*set*_) for the *k*_*th*_ visual word is defined as:

$$\begin{array}{c} \alpha \left(k\right)=\frac{1}{M\cdot (M-1)}\sum_{m=1}^{M}\sum_{l=1,l\neq m}^{M}\min(h_{Q_{m}}\left(k\right),h_{Q_{l}}\left(k\right)), \end{array}$$ (2)

where $h_{Q_{m}}$ denotes the *K*-dimensional bag-of-words histograms of the *m*_*th*_ query patch ***Q***_*m*_, *m* = 1, 2, …, *M*. A large ***α***(*k*) indicates that the similarity among the patches from the same class is high for the *k*_*th*_ visual word, i.e., the *k*_*th*_ visual word is significant to represent the patches from the class.

On the other hand, the between-class similarity ***δ***(*k*), *k* = 1, 2, …, *K*, for the *k*_*th*_ visual word is defined using the bag-of-words histograms of the patches from ***Q***_*set*_ and ***B***_*set*_ as:

$$\begin{array}{c} \delta \left(k\right)=\frac{1}{M\cdot N}\sum_{m=1}^{M}\sum_{n=1}^{N}\min(h_{Q_{m}}\left(k\right),h_{B_{n}}\left(k\right)), \end{array}$$ (3)

where $h_{B_{n}}$ denotes the bag-of-words histograms of the *n*_*th*_ background patch ***B***_*n*_, *n* = 1, 2, …, *N*. A large ***δ***(*k*) shows that the similarity among the patches from different classes is high for the *k*_*th*_ visual word, i.e., the *k*_*th*_ visual word is significant to classify the patches from different classes.

From Eq (1), the discriminative capability ***d***(*k*) for each visual word is determined. A large ***d***(*k*) comes from either a large ***α***(*k*) or a small ***δ***(*k*). Therefore, a large ***d***(*k*) indicates that the *k*_*th*_ visual word is significant not only to classify the patches from different classes but also to represent the patches from the same class.

#### 2.2.2 Testing phase

With a test microscopy image ***D***, the goal is to search for the candidate patches within ***D*** which are visually similar to at least one query patch in ***Q***_*set*_. To locate candidate patches, a voting-map based approach is exploited. Each pixel of the voting-map quantifies the confidence of an endosome centroid at the corresponding position of ***D***.

Specifically, the SIFT features are extracted from ***D*** and each of these SIFT features is assigned to a visual word generated in the training phase. To locally compare each query patch and the test microscopy image ***D***, a SIFT feature from a query patch is defined as ‘matched’ to another SIFT feature from ***D*** if both SIFT features are assigned to the same visual word. Assuming a pair of matched features ***f*** and ***g*** extracted from ***Q***_*m*_ and ***D*** is assigned to the *k*_*th*_ visual word, the location of ***f*** and its relative bias vector with respect to the centroid $c_{Q_{m}}$ of the query patch ***Q***_*m*_ can be determined geometrically by $L\left(f\right)-L\left(c_{Q_{m}}\right)$. *L*(***f***) is the center pixel position of the grid where the SIFT feature ***f*** is calculated and $L(c_{Q_{m}})$ is the center pixel position of the query patch ***Q***_*m*_. Subsequently, a possible location of the centroid ***c***_***D***_ of a candidate patch of an endosome in ***D*** is determined by $L\left(c_{D}\right)=L\left(g\right)-s*\left(L\left(f\right)-L\left(c_{Q_{m}}\right)\right)$. *L*(***g***) is the center pixel position of the grid where the SIFT feature ***g*** is calculated and *s* is the scaling factor. Since the endosomes appearing in ***D*** could be similar to a scaled version (by a scaling factor *s*) of the endosome in the query patch, we search through multiple scaling factors, i.e., $s_{1},s_{2},...,s_{n_{s}}$, within the range of [0.5, 1.5] based on the knowledge of the potential sizes of endosomes in a microscopy image.

After determining ***c***_***D***_, we calculate the pixel values of the voting-map ***V***(*s*, ***Q***_*m*_, ***D***), which is defined as an image associated with *s*, ***Q***_*m*_ and ***D***. First, all the pixel values of ***V***(*s*, ***Q***_*m*_, ***D***) are initialized as zero. For a pair of matched features being assigned to the *k*_*th*_ visual word, ***V***(*s*, ***Q***_*m*_, ***D***) is accumulated at the location ***c***_***D***_ with a value of the discriminative capability ***d***(*k*) which has been calculated in the training phase. By traversing all the matched feature pairs, the voting-map ***V***(*s*, ***Q***_*m*_, ***D***) is generated. To integrate the information from multiple query patches, a general voting-map ***V***(*s*, ***Q***_*set*_, ***D***) associated with *s*, ***Q***_*set*_, and ***D*** can be determined as the summation of the voting-map associated with every query patch in ***Q***_*set*_:

$$\begin{array}{c} V\left(s,Q_{set},D\right)=\sum_{m=1}^{M}V(s,Q_{m},D). \end{array}$$ (4)

To make the notation less clumsy, the notation for ***V***(*s*, ***Q***_*set*_, ***D***) is simplified as ***V***_*s*_. ***V***_*s*_(*x*, *y*) denotes the value of the pixel at the *x*_*th*_ row and the *y*_*th*_ column of ***V***_*s*_. After repeating the same procedure for all the scaling factors $s_{1},s_{2},...,s_{n_{s}}$, *n*_*s*_ voting-maps $V_{s_{1}},V_{s_{2}},...,V_{s_{n_{s}}}$ are generated. A final voting-map ***V***_*final*_ is determined by selecting the optimal scaling factor for each pixel position. Specifically, for the position (*x*_*v*_, *y*_*v*_), the optimal scaling factor $s_{\left(x_{v},y_{v}\right)}^{*}$ is determined by selecting the scaling factor which produces the maximum pixel value of $V_{s_{1}},V_{s_{2}},...,V_{s_{n_{s}}}$ at (*x*_*v*_, *y*_*v*_), i.e.,:

$$\begin{array}{c} s_{\left(x_{v},y_{v}\right)}^{*}=\arg\max_{s\in \{s_{1},s_{2},...,s_{n_{s}}\}}V_{s}\left(x_{v},y_{v}\right). \end{array}$$ (5)

The value of the final voting-map ***V***_*final*_ at (*x*_*v*_, *y*_*v*_) is determined as:

$$V_{final}\left(x_{v},y_{v}\right)\overset{\Delta }{=}V_{s_{\left(x_{v},y_{v}\right)}^{*}}\left(x_{v},y_{v}\right).$$ (6)

**Fig 5:**
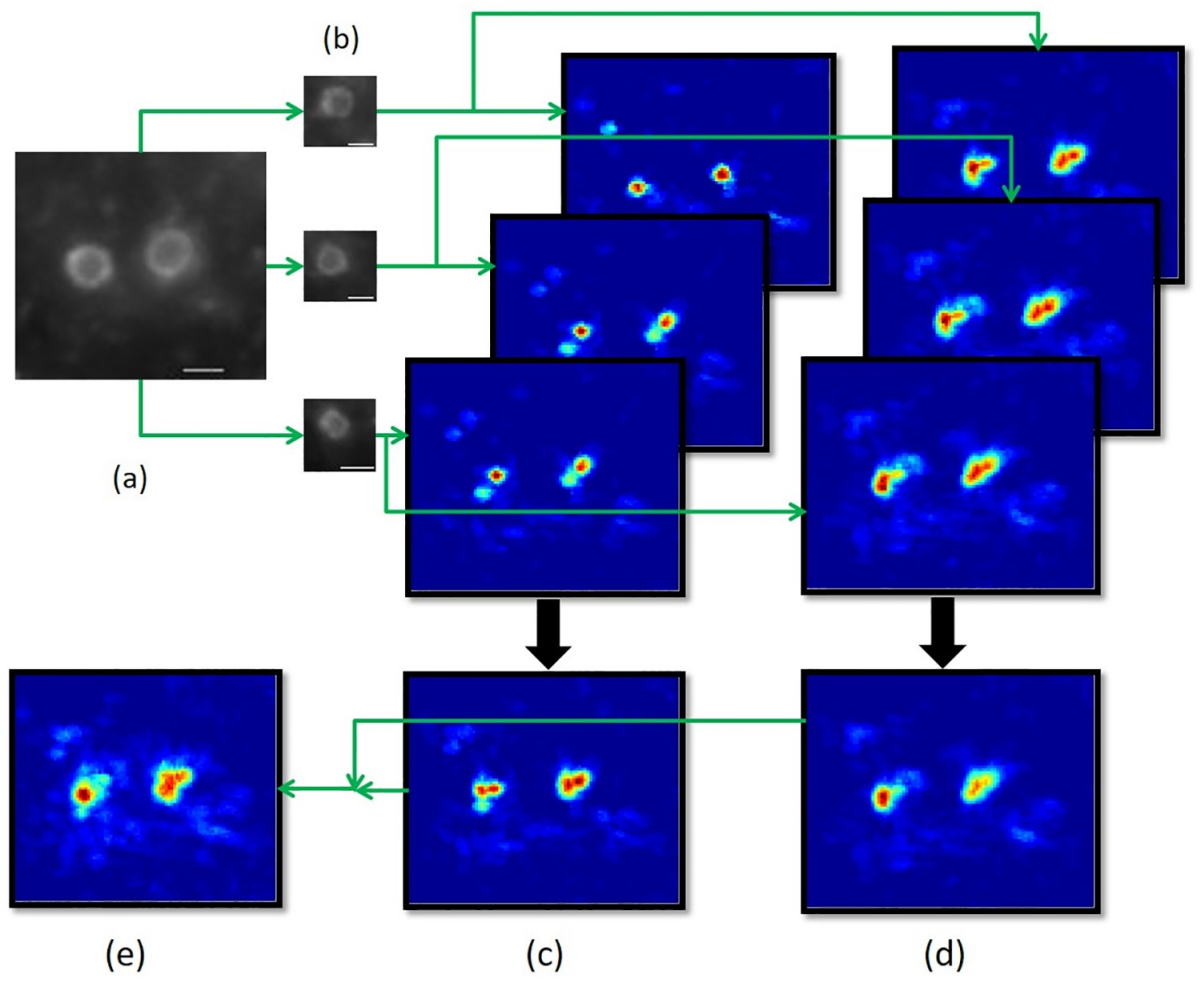
A diagram illustrating the procedure of voting-map generation. Three query patches and two scaling factors, i.e., 0.8 and 1, are adopted in this example. (a) The test microscopy image. (b) The three query patches. (c) The generation of the voting-maps with the scaling factor set at 0.8. For each query patch, a voting-map is generated by local feature matching between the query patch and the test image. Then a voting-map associated with the scaling factor of 0.8 is obtained by summing the three voting-maps. (d) The generation of the voting-maps with the scaling factor set at 1. (e) The final voting-map is determined by selecting the optimal scaling factor for each pixel position as described in the text. Scale bar = 1 *μ*m.

The final voting-map ***V***_*final*_ and the corresponding $s_{\left(x_{v},y_{v}\right)}^{*}$ for each pixel in ***V***_*final*_ will be adopted in the identification stage. Fig 5 illustrates the voting-map generation procedure.

### 2.3 Identification stage

After the localization stage, we generate the voting-map ***V***_*final*_ and the optimal scaling factor corresponding to each pixel in ***V***_*final*_. Candidate patches of endosomes centered at the locations with high voting-map pixel values can be obtained from the test microscopy image. The identification stage is proposed to train a support vector machine to identify endosome patches and rule out endosome-like background patches. The identification stage also consists of a training phase and a testing phase.

#### 2.3.1 Training phase

In the training phase, a binary support vector machine (SVM) is trained to classify endosome patches from background pattern patches. Here, we treat endosomes as the positive class and background patterns as the negative class. The same query and background pattern patches used in the localization stage are adopted as the training patches in the identification stage.

To represent each training patch, we apply a modified bag-of-words model where locality-constrained linear coding (LLC) [20] is adopted. The LLC modifies the way that SIFT features are assigned to the visual words. Given a SIFT feature ***f***, the original feature assignment is to select the nearest visual word to ***f*** in Euclidean distance. This approach produces large quantization errors. To reduce the quantization errors, LLC encodes a SIFT feature with multiple visual words [20]. Specifically, LLC first selects *C* nearest visual words for ***f*** among all the visual words to form a local base ***B***_***f***_ = [***b***_**1*f***_, ***b***_**2*f***_, …***b***_***Cf***_]. Then it solves the following optimization problem to determine a code vector ***c***_***f***_ = [*c*_*f*1_, *c*_*f*2_, …, *c*_*fC*_]^*T*^ for ***f***:

$$\begin{array}{c} \begin{array}{c} \min_{c_{f}}{∥f-B_{f}c_{f}∥}^{2},\\ st.\;1^{T}c_{f}=1, \end{array} \end{array}$$ (7)

where **1** denotes a column vector with each entry value as 1. With each SIFT feature represented by an LLC code, the modified bag-of-words representation for the patch is determined by max-pooling these LLC codes, i.e., selecting the component-wise maximum value for each LLC code [20]. A binary SVM with a linear kernel is trained to classify the training patches represented by the modified bag-of-words histograms. The details of determining the optimal SVM hyperplane by solving a quadratic programming problem can be seen in [25].

**Remark**. *In the proposed method, we adopt vector quantization to encode the SIFT features with respect to the visual words in the localization stage and adopt LLC encoding in the identification stage. For the localization stage, we aim to measure the similarity between two SIFT features and therefore comparing the nearest visual word for these two features is a starightforward approach. For the identification stage, the goal is to build a dicriminative representation for a patch where using multiple visual words to represent each SIFT feature extracted from the patch has been proved more effective in* [20].

#### 2.3.2 Testing phase

**Fig 6:**
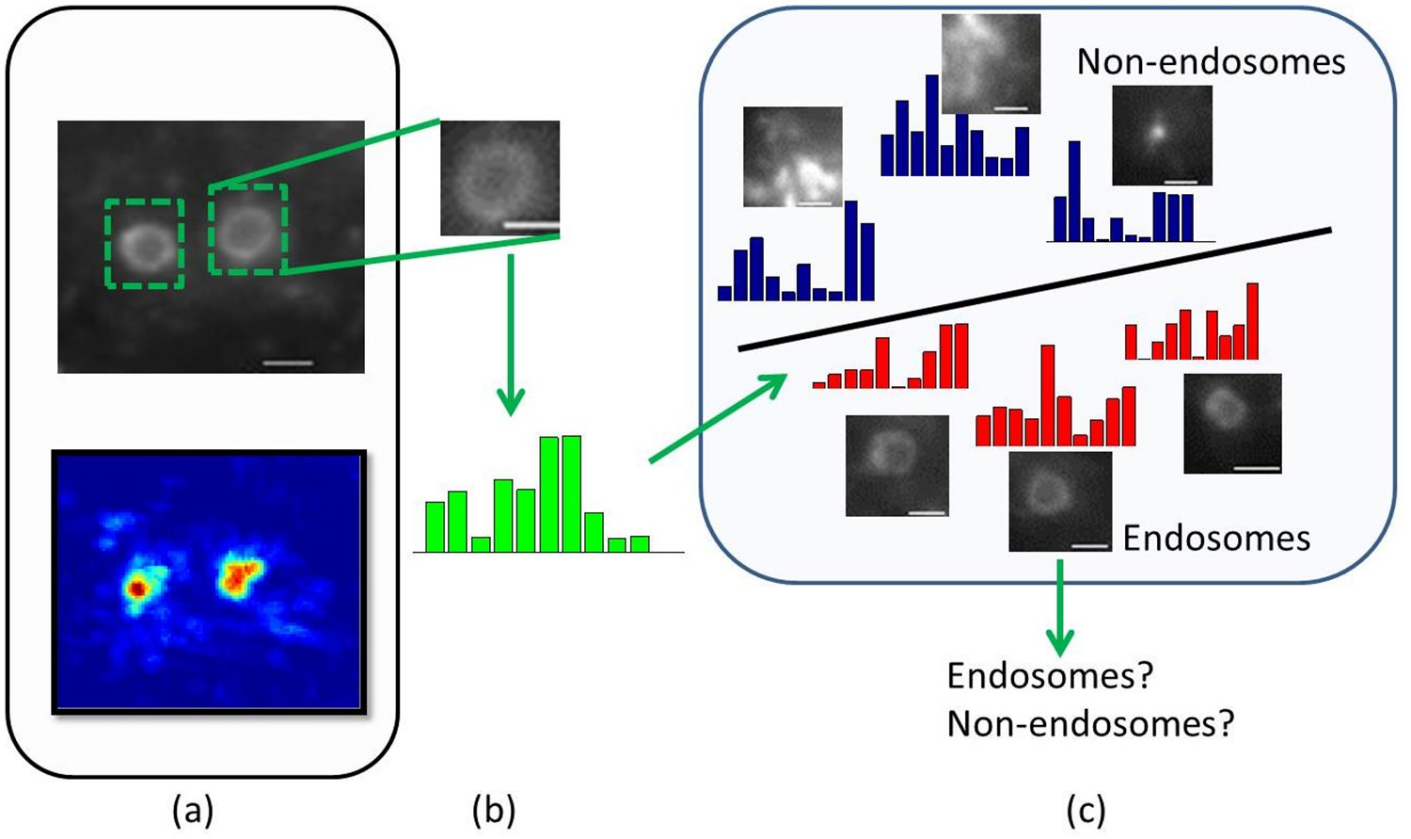
A diagram illustrating the identification stage using SVM. (a) After the voting-map thresholding and non-maximum suppression, the candidate patches of endosomes are obtained. (b) Each candidate patch is represented by the LLC-based bag-of-words histogram. (c) The histogram is classified by the SVM to determine whether the patch contains an endosome or a background pattern. Scale bar = 1 *μ*m.

In the testing phase, the voting-map is first normalized to the range [0, 1] and a threshold *γ* is set for the voting-map ***V***_*final*_ to rule out those locations with very low values. Consequently, the thresholded voting-map only retains the pixels whose values are larger than *γ* and sets other pixels to zero. Subsequently, the candidate patches of endosomes are determined. Specifically, for a non-zero pixel location {*x*_*o*_, *y*_*o*_} in the thresholded voting-map, the optimal scaling factor $s_{\left(x_{o},y_{o}\right)}^{*}$ which is recorded in the localization stage is retrieved. Assuming the width and height of each query patch being *w* and *h*, respectively, a candidate patch can be obtained and parameterized as [*x*_min_, *y*_min_, *x*_max_, *y*_max_], where (*x*_min_, *y*_min_) and (*x*_max_, *y*_max_) denote the pixel positions of the upper-left and the bottom-right corners of the candidate patch, respectively. They are calculated as:

$$\begin{array}{c} \begin{array}{c} x_{\min}=\lceil x_{o}-\frac{1}{2}s_{\left(x_{o},y_{o}\right)}^{*}\times w\rceil ,y_{\min}=\lceil y_{o}-\frac{1}{2}s_{\left(x_{o},y_{o}\right)}^{*}\times h\rceil ,\\ x_{\max}=\lfloor x_{o}+\frac{1}{2}s_{\left(x_{o},y_{o}\right)}^{*}\times w\rfloor ,y_{\max}=\lfloor y_{o}+\frac{1}{2}s_{\left(x_{o},y_{o}\right)}^{*}\times h\rfloor , \end{array} \end{array}$$ (8)

where ⌈.⌉ is the ceiling function which outputs the smallest integer larger than or equal to the input real number, and ⌊.⌋ is the floor function which outputs the greatest integer which is smaller than or equal to the input real number. In this way, a candidate patch [*x*_min_, *y*_min_, *x*_max_, *y*_max_] and its voting-map value ***V***_***final***_(*x*_*o*_, *y*_*o*_) is retrieved. Then, the non-maximum suppression (NMS) algorithm [26] is applied to handle the duplicated detection, i.e., for multiple candidate patches containing the same target, the patch with the highest voting-map value is selected and the others are removed (suppressed). Finally, the remaining candidate patches are classified by the SVM from the training phase as shown in Fig 6.

Algorithm 1 summarizes the overall procedure for the detection of ring-like endosomes in a test microscopy image. The algorithm consists of the localization stage and identification stage. The test microscopy image is fed to the two stages in sequence and the detection of endosomes is automatically determined.

**Algorithm 1** The procedure for the detection of endosomes in a test microscopy image.

***D***: the test image.

***Q***_*m*_: the *m*_*th*_ query patch in the query set ***Q***_*set*_.

*M*: the number of query patches.

*K*: the number of visual words.

*n*_*s*_: the number of the elements in the scaling factor set $s=\{s_{1},s_{2},...,s_{n_{s}}\}$.

*L*(***f***): the location of the SIFT feature ***f***.

*L*(***g***): the location of the SIFT feature **g**.

*L*(***c***_***D***_): the location of the centroid of the test image ***D***.

$L(c_{Q_{m}})$: the location of the centroid of the query patch ***Q***_*m*_

**Initialization**: Initialize the pixel values for the voting-maps $V_{s_{1}},V_{s_{2}},...,V_{s_{n_{s}}}$ as zeros.

**Localization Stage**:

**for** *s* = *s*_1_ to $s_{n_{s}}$ **do**

 **for** visual word *k* = 1 to *K* **do**

  **for** query patch *m* = 1 to *M* **do**

   **for** local features ***f*** ∈ ***Q***_*m*_ and ***g*** ∈ ***D*** **do**

    **if** ***f*** and ***g*** are both assigned to the *k*_*th*_ visual word **then**

$L\left(c_{D}\right)=L\left(g\right)-s*\left(L\left(f\right)-L\left(c_{Q_{m}}\right)\right)$.

     ***V***_*s*_(*L*(***c***_***D***_)) = ***V***_*s*_(*L*(***c***_***D***_)) + ***d***(*k*).

    **end if**

   **end for**

  **end for**

 **end for**

**end for**

**for** Each location {*x*_*v*_, *y*_*v*_} **do**

 Determine the optimal scaling factor $s_{\left(x_{v},y_{v}\right)}^{*}$ by (5).

 Determine the values of the final voting-map ***V***_*final*_ by (6).

**end for**

**Identification Stage**:

Normalize the values of the final voting-map ***V***_*final*_ into the range [0, 1] and threshold the final voting-map by a given threshold *γ*.

**for** Each location {*x*_*o*_, *y*_*o*_} with non-zero thresholded voting-map value ***V***_*final*_(*x*_*o*_, *y*_*o*_) **do**

 The optimal scaling factor $s_{\left(x_{o},y_{o}\right)}^{*}$ and ***V***_*final*_(*x*_*o*_, *y*_*o*_) are retrieved.

 A candidate patch ***P*** centered at {*x*_*o*_, *y*_*o*_} is determined by (2.3.2).

**end for**

Apply the non-maximum suppression (NMS) algorithm to remove the duplicated detection patches.

**for** Each candidate patch ***P* do**

 Calculate the bag-of-words histogram from ***P***.

 Apply the SVM to classify the histogram and determine if ***P*** contains an endosome or not.

**end for**

## 3 Experiments

In this section, we first describe the dataset in Subsection 3.1. In Subsection 3.2, several metrics to evaluate the detection performance are introduced. The experimental protocol and parameter settings are specified in Subsection 3.3. The experimental results are presented and analyzed in Subsection 3.4.

The competing methods include two one-stage supervised learning methods and one unsupervised learning method. For the supervised learning methods, one is the AdaBoost classifier trained on Haar-like features (AB-Haar). This method was originally proposed in [16] to systematically build a face detector and was extended for the detection of molecular particles in live cells in [17]. The second is a linear discriminant analysis (LDA) model trained on simple pixel features (LDA-Pixel) which was proposed in [13] for spot detection in microscopy images. For the competing unsupervised method, we choose the method proposed in [14] for cell nuclei detection mainly based on image morphological operations and fast radial symmetric transform (MO-FRST).

### 3.1 Dataset description

The detection performance of the compared methods is evaluated using the experimental microscopy images of human myeloid endothelial cells generated in our laboratory. To generate the dataset, the human myeloid endothelial cells were maintained, transfected with DNA plasmids that express green fluorescent protein-tagged neonatal Fc receptor (FcRn-GFP), plated in glass-bottom dishes and imaged as described in [2, 27]. The images were acquired using a Zeiss AxioObserver.Z1 microscopy with widefield arc lamp illumination, a 63X 1.4NA Plan Apochromat oil immersion objective, a GFP-specific filterset (GFP-3035D-000, Semrock) and a monochromatic CCD camera (Orca ER, Hamamatsu). The dataset consists of two groups of microscopy images with different total magnifications, i.e., 100.8X and 157.5X, respectively. In total, there are 50 microscopy images which contain 1346 endosomes annotated by the analysts. Table 1 summarizes the specific information of the dataset.

**Table 1:** The dataset information.

Total magnification	100.8X	157.5X
# of microscopy images	30	20
# of annotated endosomes	765	581

### 3.2 Performance metrics

The detection performance is evaluated based on an overlay of the detected patches and the ground truth patches annotated by the analysts. A detected patch is defined as correct if the area of overlap (*α*_0_) between the detected patch *b*_*p*_ and the ground truth patch *b*_*gt*_ exceeds 50%, where *α*_0_ is defined as

$$\begin{array}{c} \alpha_{0}=\frac{area(b_{p}\cap b_{gt})}{area(b_{p}\cup b_{gt})}, \end{array}$$ (9)

where *b*_*p*_ ∩ *b*_*gt*_ denotes the intersection of the detected and ground truth patches and *b*_*p*_ ∪ *b*_*gt*_ denotes their union [28]. Based on this criterion, three basic performance statistics, namely *N*_*FN*_, *N*_*TP*_ and *N*_*FP*_ are determined. *N*_*FN*_ is the number of false negative detection, i.e., the number of patches which are in the ground truth but not detected by the method. *N*_*TP*_ is the number of true positive detection, i.e., the number of patches which are in the ground truth and also detected by the method. *N*_*FP*_ is the number of false positive detection, i.e., the number of patches which are detected by the method but not shown in the ground truth.

Using these three statistics, several performance metrics are defined. For classifier based detection methods, the confidence of each detected patch is quantified by a score output from the classifier, e.g., posterior probabilities. By setting thresholds to the confidence scores, we can plot (using log-log plots) the miss rate (MR) against the false positives per image (FPPI) curves [29, 30] to show the overall detection performance, where the miss rate (MR) is calculated by $MR=\frac{N_{FN}}{N_{FN}+N_{TP}}$ and the false positives per image (FPPI) is the average value of *N*_*FP*_ over the test microscopy images. Specifically, given a threshold, a pair of values of the false positives per image (FPPI) and the corresponding miss rate (MR) are determined. By varying the threshold, an MR-FPPI curve can be plotted. A better detection method produces a lower miss rate and a smaller number of the false positive detection and therefore its MR-FPPI curve stays closer to the left-bottom corner [29].

Quantitatively, four performance metrics are calculated, namely the log-average miss rate, the precision, the recall and the *F*_1_ score. The log-average miss rate is defined based on the MR-FPPI curve. It is calculated by averaging miss rate at nine FPPI rates evenly spaced in log-space in the range 10^−2^ to 10^0^ [29]. The definitions of the precision (P), the recall (R) and the *F*_1_ score are as follows:

$$\begin{array}{c} P=\frac{N_{TP}}{N_{FP}+N_{TP}},R=\frac{N_{TP}}{N_{FN}+N_{TP}},F_{1}=\frac{2\times P\times R}{P+R}. \end{array}$$ (10)

The precision is the ratio of the number of the correctly detected patches to that of all the detected patches. The recall is the ratio of the number of the correctly detected patches to that of all the ground truth patches. The *F*_1_ score combines the information from both the precision and the recall. The values of log-average miss rate, the precision, the recall and the *F*_1_ score are within [0, 1]. A better detection method produces higher values for the precision, the recall and the *F*_1_ score while a lower value for the log-average miss rate.

### 3.3 Experimental protocol and parameter settings

We conduct 500 rounds of independent experiments to evaluate the detection performance of the compared methods. For each round, we randomly select 20% of the microscopy images for training and the remaining 80% of the images for testing. This experimental protocol is consistently applied to the two groups of microscopy images with different total magnifications, respectively. All the performance metrics are calculated as the average (with standard deviations) of the 500 rounds of experiments.

For the proposed method, in the localization stage, we select 50 query patches of endosomes together with 200 patches of the background patterns to determine the voting-map. The size of these patches is set at 45 × 45 (pixels). For bag-of-words model generation, each training patch (and each test microscopy image) is divided into 16 × 16 (pixels) overlapping grids and the grid space of two neighboring grids is set at 2 pixels. A SIFT feature is extracted from each grid. The number of visual words for the bag-of-words dictionary is set at 400. The range for the scaling factor searching is [0.5, 0.6, …, 1.5] and therefore *n*_*s*_ is set at 11. The threshold *γ* for the normalized voting-map is set at 0.2. For locality-constrained linear coding (LLC) [20], the number of visual words selected to encode a SIFT feature is set at 5. For SVM training, the regularization parameter *C* of the SVM is selected through a 5-fold cross validation within the range of [2^−8^, 2^−7^, …, 2^7^, 2^8^].

For the two competing supervised methods, namely, AB-Haar and LDA-Pixel, the positive and negative patches are the same as those adopted in the proposed method. For the AB-Haar method, each training patch is represented by the Haar-like features adopted in [13]. For the LDA-Pixel method, each training patch is represented by a feature vector generated through concatenating the horizontal rows of pixels [13]. The sliding window size is set at 45 × 45 (pixels) and the two neighboring sliding windows have 14 pixels in overlapping for both horizontal and vertical directions.

For the unsupervised method, i.e., MO-FRST [14], the searching range of the radius of FRST transformation is set at [16, 18, …, 30] (pixels) to cover the possible radius of an endosome. The radial strictness parameter in FRST is set at 2, the scaling factor is set at *k*_*ρ*_ = 10 and the size of the pixels for the non-maximum suppression (NMS) is set at 20 [14].

### 3.4 Experimental results and analysis

**Fig 7:**
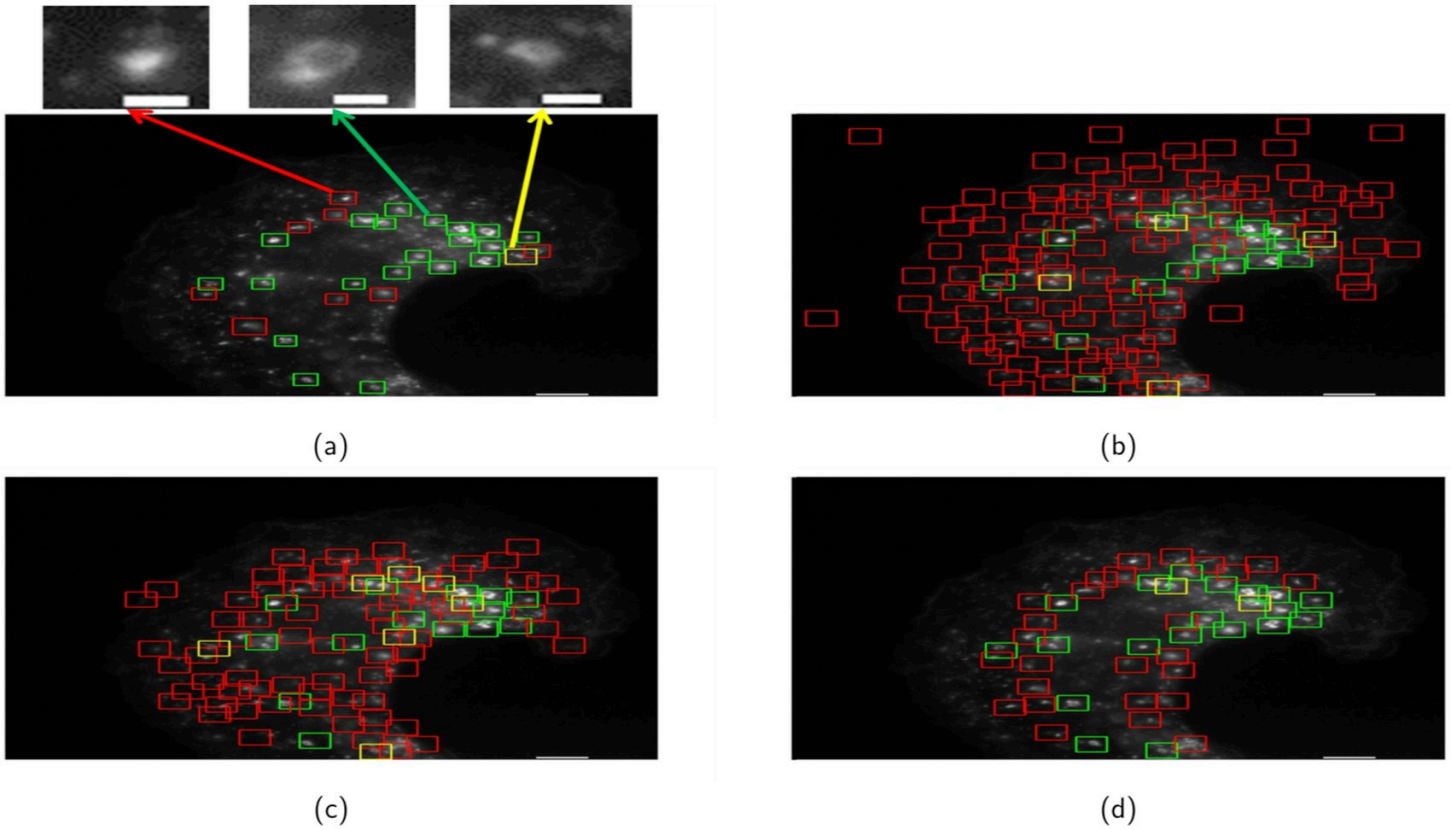
The detection results for an example microscopy image with the total magnification 100.8X. (a) the proposed method, (b) AB-Haar, (c) Pixel-LDA, (d) MO-FRST. The true positives, the false positives and the false negatives are represented by green, red, and yellow patches, respectively in the color version of this paper. In (a), we show three enlarged patches which are a false positive (indicated by a red arrow), a true positive (green arrow) and a false negative (yellow arrow). The scale bar for the large microscopy images is 5 *μ*m and that for the three small patches shown in (a) is 1 *μ*m.

**Fig 8:**
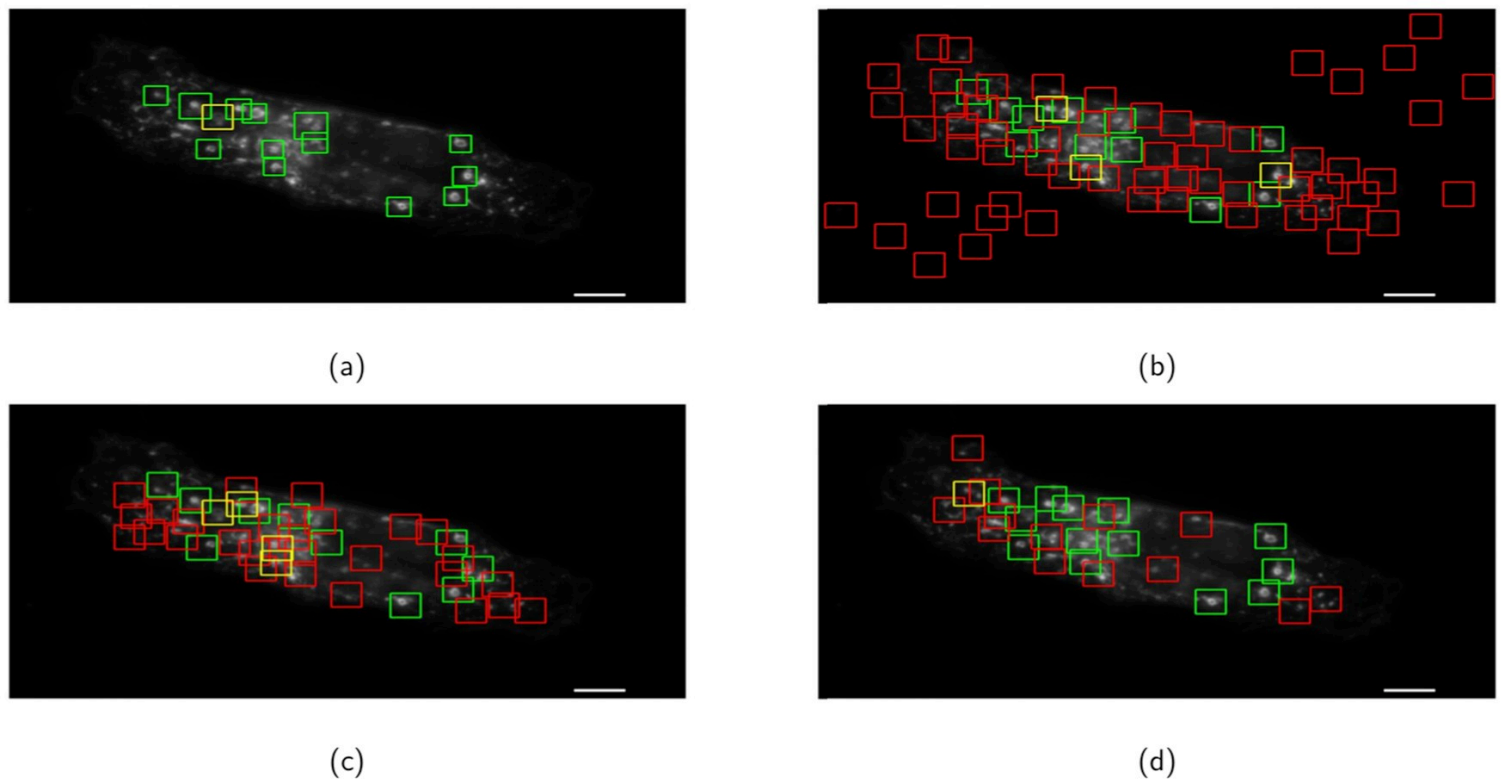
The detection results for an example microscopy image with the total magnification 157.5X. (a) the proposed method, (b) AB-Haar, (c) Pixel-LDA, (d) MO-FRST. Here, the notations are the same as Fig 7. The true positives, the false positives and the false negatives are represented by green, red, and yellow patches, respectively. Scale bar = 5 *μ*m.

Figs 7 and 8 show the qualitative detection performance for two test microscopy images with different total magnifications. As shown in Figs 7a and 8a, the proposed method approximately localizes each endosome at the center of the detected patch and automatically determines the size of each detected patch. However, Both AB-Haar and LDA-Pixel methods suffer from the “mismatch” between the sliding window and the endosome (see Figs 7b and 7c, 8b and 8c). The MO-FRST can localize the centroid of all the circular structures but fail to further distinguish the endosomes and background patterns that are circularly shaped (see Figs 7d and 8d). The proposed method produces the highest number of true positive detection and the lowest number of false positive and false negative detection among all the compared methods. It is also noted that the proposed method can better handle the detection of the endosomes which stay very close to or even touching each other since the proposed localization stage locally compares the SIFT features. Hence, even when an endosome only partially appears, there are still some local features being determined as matched to the local features extracted from the query patches. However, it is observed that the performance of the competing methods significantly deteriorate in this scenario.

**Fig 9:**
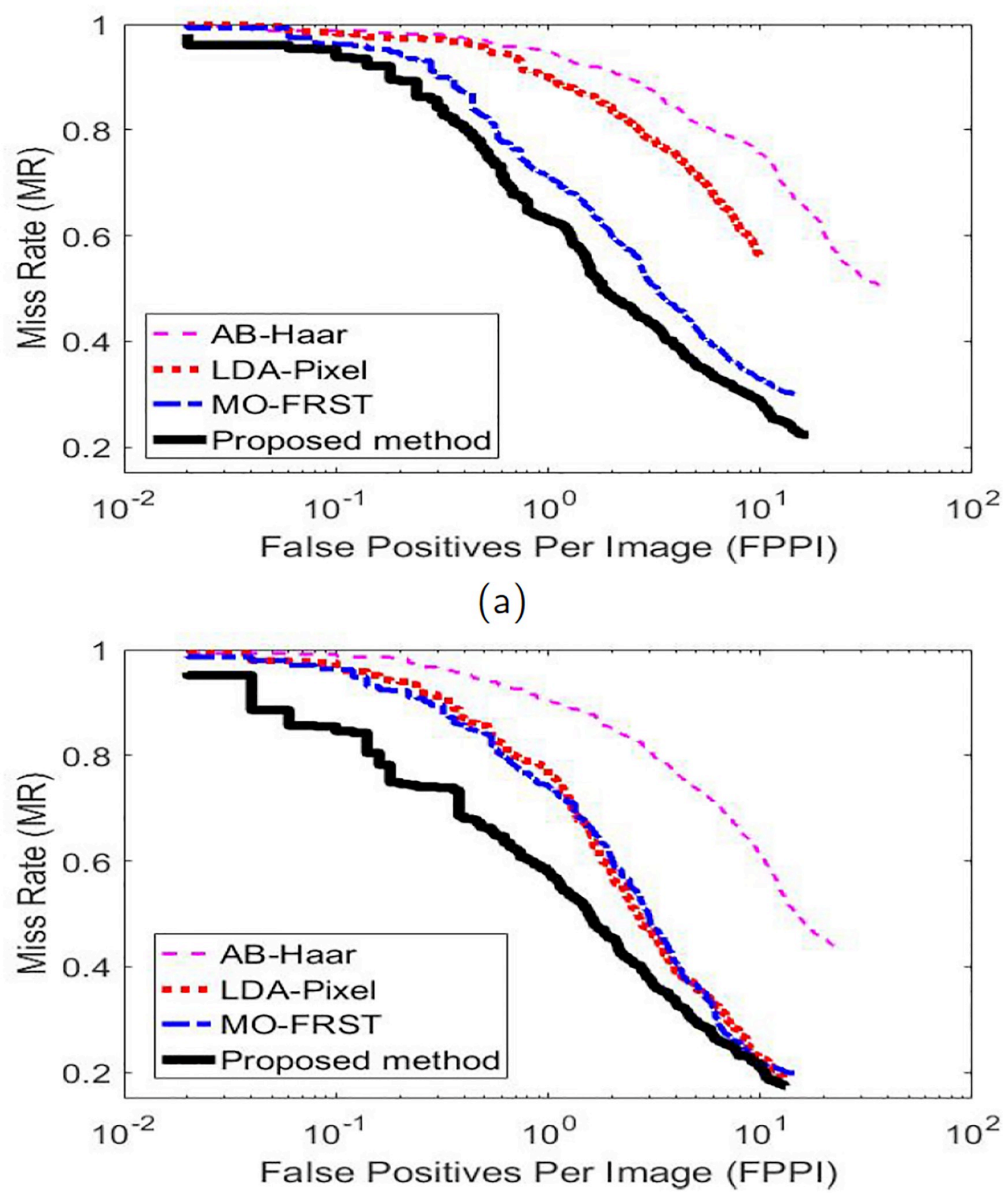
The MR-FPPI curves of the compared methods. (a) the microscopy images with the total magnification of 100.8X. (b) the microscopy images with the total magnification of 157.5X.

Quantitatively, the detection performance of the compared methods is shown by the MR-FPPI curves and the four metrics introduced in Section 3.2. Fig 9a and 9b show the MR-FPPI curves of the compared methods for the two groups of microscopy images with different total magnifications, respectively. Each curve is plotted based on one typical trial out of 500 rounds of experiments. It is noted that we have similar observations from the MR-FPPI curves of different experimental trials. It is observed that the proposed method outperforms the competing methods as its MR-FPPI curve stays the closest to the left-bottom corner. Table 2 presents the quantitative performance metrics of the compared methods (the results for the best method are bolded). It is observed from the table that the proposed method significantly outperforms all the competing methods in terms of producing the lowest log-average miss rate and the highest precision, recall and *F*_1_ score, respectively. This indicates the proposed method produces more accurate detection of endosomes and lower missing detection than the competing methods.

**Table 2:** Average performance metrics (and standard deviations) for the compared methods.

Methods	Log-average miss rate	Precision	Recall	*F*_1_ score
The images with a total magnification of 100.8X				
AB-Haar [17]	0.529 (0.006)	0.296 (0.012)	0.660 (0.007)	0.409 (0.011)
LDA-Pixel [13]	0.344 (0.005)	0.615 (0.025)	0.636 (0.008)	0.625 (0.010)
MO-FRST [14]	0.269 (0.009)	0.519 (0.011)	0.761 (0.005)	0.617 (0.007)
Proposed method	**0.138 (0.007)**	**0.812 (0.009)**	**0.807 (0.005)**	**0.773 (0.006)**
The images with a total magnification of 157.5X				
AB-Haar [17]	0.457 (0.022)	0.338 (0.009)	0.695 (0.007)	0.455 (0.008)
LDA-Pixel [13]	0.235 (0.023)	0.475 (0.013)	0.846 (0.009)	0.608 (0.011)
MO-FRST [14]	0.241 (0.015)	0.441 (0.018)	0.833 (0.005)	0.577 (0.016)
Proposed method	**0.097 (0.013)**	**0.819 (0.012)**	**0.850 (0.015)**	**0.770 (0.009)**

More specifically, for those one-stage supervised methods, since ring-like endosomes are not just simple “bright structures” in the dark background like spots, features like Haar-like features or pixel intensities adopted for bright spot detection might not be sufficiently discriminative to describe the micro-structures of endosomes. On the other hand, the proposed method extracts SIFT features summarizing local gradients which can robustly capture the distinct local structures of endosomes despite of the influences of noise and illuminations in the microscopy images. Therefore, the proposed method outperforms the two one-stage supervised methods by extracting more discriminative features. Moreover, the proposed two-stage detection structure utilizes both local and global information from the endosome patches and the background pattern patches. On the one hand, searching for matched SIFT features in the localization stage provides the location of each candidate endosome. On the other hand, the adoption of the bag-of-words model modified by LLC to represent the patches for SVM training applies the global information of each training patch as a whole to identify endosomes. Hence, the proposed method outperforms the one-stage supervised detection methods whose detection performance highly relies on the classifiers as only the global information is incorporated.

For the comparison with the unsupervised method, MO-FRST [14] was specifically formulated to detect cell nuclei which are circular and symmetrical “bright structures”. However, endosomes are only approximately circular and symmetrical. Moreover, they have ring-like textures. MO-FRST produces high false positive detection since it only captures all circular and symmetrical patterns but fails to further identify the ring-like structures. In contrast, the proposed method exploits distinct textures of ring-like endosomes in both stages and hence produces much lower false positive detection rate.

## 4 Conclusion

In this paper, we have proposed a two-stage method for automatically detecting ring-like endosomes in fluorescent microscopy images. The proposed method is a supervised method and therefore it does not require much prior knowledge as unsupervised methods do. Once the training phase in the two stages is performed off-line, the method can achieve fully automated detection of ring-like endosomes. Essentially, the method exploits local SIFT feature matching to locate candidate endosome patches and then identifies each of the candidate patches using a pretrained SVM from a classification perspective. The experiments on the real microscopy images show that the proposed method can produce significantly better detection results compared with several competing methods in terms of multiple detection performance metrics.
